# Distribution of MRI-derived T2 values as a biomarker for in vivo rapid screening of phenotype severity in *mdx* mice

**DOI:** 10.1371/journal.pone.0310551

**Published:** 2024-09-19

**Authors:** Emily A. Waters, Chad R. Haney, Lauren A Vaught, Elizabeth M. McNally, Alexis R. Demonbreun

**Affiliations:** 1 Chemistry of Life Processes Institute and Biomedical Engineering, Northwestern University Feinberg School of Medicine, Chicago, IL, United States of America; 2 Center for Genetic Medicine, Northwestern University Feinberg School of Medicine, Chicago, IL, United States of America; 3 Department of Pharmacology, Northwestern University Feinberg School of Medicine, Chicago, IL, United States of America; The University of Texas Rio Grande Valley, UNITED STATES OF AMERICA

## Abstract

**Background:**

The pathology in Duchenne muscular dystrophy (DMD) is characterized by degenerating muscle fibers, inflammation, fibro-fatty infiltrate, and edema, and these pathological processes replace normal healthy muscle tissue. The *mdx* mouse model is one of the most commonly used preclinical models to study DMD. Mounting evidence has emerged illustrating that muscle disease progression varies considerably in *mdx* mice, with inter-animal differences as well as intra-muscular differences in pathology in individual *mdx* mice. This variation is important to consider when conducting assessments of drug efficacy and in longitudinal studies. We developed a magnetic resonance imaging (MRI) segmentation and analysis pipeline to rapidly and non-invasively measure the severity of muscle disease in *mdx* mice.

**Methods:**

Wildtype and *mdx* mice were imaged with MRI and T2 maps were obtained axially across the hindlimbs. A neural network was trained to rapidly and semi-automatically segment the muscle tissue, and the distribution of resulting T2 values was analyzed. Interdecile range and Pearson Skew were identified as biomarkers to quickly and accurately estimate muscle disease severity in mice.

**Results:**

The semiautomated segmentation tool reduced image processing time approximately tenfold. Measures of Pearson skew and interdecile range based on that segmentation were repeatable and reflected muscle disease severity in healthy wildtype and diseased *mdx* mice based on both qualitative observation of images and correlation with Evans blue dye uptake.

**Conclusion:**

Use of this rapid, non-invasive, semi-automated MR image segmentation and analysis pipeline has the potential to transform preclinical studies, allowing for pre-screening of dystrophic mice prior to study enrollment to ensure more uniform muscle disease pathology across treatment groups, improving study outcomes.

## Introduction

Duchenne muscular dystrophy (DMD) is an inherited muscle wasting disease caused by loss of function mutations in dystrophin [1,2]. Dystrophin, a large protein that links the cytoskeleton with the extracellular matrix, is encoded by the *DMD* gene. In the absence of dystrophin, the muscle membrane becomes easily disrupted which causes muscles to degenerate, and in its place, the intact muscle is replaced by fibrosis and fat, as well as immune infiltrate. The dystrophin-deficient *mdx* mouse model on the C57Bl/10 background harbors a naturally occurring mutation in exon 23 of dystrophin resulting in a premature stop codon [3,4]. It is the model most commonly used in preclinical therapeutic trials as it recapitulates many of the same pathological features seen in humans with DMD [5]. Although the *mdxB10* mouse produces no detectable dystrophin protein, this model exhibits milder histological and functional deficits than humans with DMD. A more severe mouse model referred to as *mdxD2* was generated through backcrossing the *mdx*C57Bl/10 model over 5 generations onto the DBA/2J strain [6]. The muscle pathology in *mdxD2* mice recapitulates additional features of human DMD pathology with increased areas of muscle damage consisting of increased fibrofatty deposition, myofiber necrosis, and immune infiltrate. Along with these findings, *mdxD2* mice exhibit impaired repair capacity and greater functional deficits compared to the *mdx*C57Bl/10 model [7–11].

The availability of multiple mouse models of DMD has allowed for extensive preclinical studies to evaluate potential therapeutics. With preclinical testing occurring across multiple animal colonies, there have been efforts to standardize procedures to increase the reliability and translatability of endpoint measures (TREAT-NMD.org protocols). It is well known that the *mdx* model displays a wide range of both intra-animal variability and inter-animal variability [12–15]. For example, *mdx* littermates can exhibit virtually no muscle damage or fibrosis in one subject, while another is severely affected. Moreover, within the same animal one muscle may have extensive pathology while the contralateral muscle is minimally affected. This inherent variation can contribute to poor assay sensitivity and necessitates large cohorts of animals to achieve the statistical power required to show treatment effects [16]. Spurney et al showed that quantitative histology measures of degeneration and regeneration, and creatine kinase showed high variance in the *mdx* model. Evans blue dye uptake, as a measure of muscle membrane leak, also showed both animal to animal variability and intra-animal variability across studies [17].

Understanding the mitigating factors that can influence preclinical outcome measures is critical when designing and evaluating therapeutic efficacy studies. Environmental factors such as cage design, light/dark cycle, food, sex, age, and weight can be more easily controlled than biological factors. Non-invasive imaging techniques such as T_2_-weighted magnetic resonance imaging (MRI) can provide information on *in vivo* muscle tissue health at the outset of a study, without the need for tissue sampling or sacrifice. T_2_ is a property of tissue relaxation in the presence of a magnetic field; it tends to be high in the presence of free fluid, and is often abnormally elevated in the presence of inflammation, edema, and necrosis [18]. Assigning animals to treatment groups based on visual and qualitative estimates of disease severity risks introducing bias, while a labor- and time-intensive processing protocol is not feasible to implement at the beginning of a large study. Further, it can be challenging to identify a numerical summary statistic that reflects the qualitative differences readily observed between imaging datasets. For example, in datasets with small, localized regions of signal change, signal averaging can obscure differences due to the much larger areas of unchanged tissue. Indeed, previous efforts to use average T_2_ values as an imaging biomarker of disease progression in *mdx* mice have been hampered by high variance that has been interpreted as a lack of significant differences between timepoints or groups, but more likely reflects the inadequacy of bulk averaging as a summary statistic [18]. We hypothesize that the distribution of muscle T_2_ values reflects features of the underlying disease, and propose a rapid high-throughput screening method that combines MR imaging, semi-automated segmentation, and quantitative analysis with meaningful summary statistics to estimate disease severity *in vivo*, balance treatment groups, and *a priori* exclude animals with unusually high or low disease burdens.

## Methods

### Animals

The 6–7 weeks old WTC57Bl/6, *mdxB10* and *mdxD2* male mice were purchased from the Jackson Laboratory (stock 000664, 001801, and 013141, respectively) and imaged at 8 weeks of age. Mice were housed in a specific pathogen–free facility on a 12-hour light/12-hour dark cycle, fed ad libitum and sacrificed in accordance with the Northwestern University’s Institutional Animal Care and Use Committee regulations and the NIH Guide for the Care and Use of Laboratory Animals to minimize distress.

### MR image acquisition

Mice were anesthetized in an induction chamber with inhaled isoflurane and then transferred to a dedicated imaging bed with a nosecone to deliver continuous isoflurane. Each mouse was positioned prone with legs tucked beneath the abdomen to reduce susceptibility artifacts. Respiration was monitored using a pressure sensitive pillow and body temperature was maintained with a warm water circulating jacket placed under the animal. MRI was performed on a 9.4T Bruker Biospec 9430 (Bruker Corporation, Billerica, MA, USA) with a 30 cm bore and 12 cm gradient insert, running Paravision 6.0.1. Each mouse was imaged using a 40 mm quadrature volume coil (Bruker) operating in transmit/receive mode. After localizer images were acquired, a T_2_ map was acquired using a spin echo sequence (Multi Slice Multi Echo, MSME) oriented axially and centered at the mid-calf. The following parameters were used: TR = 4000 ms, TE = 9–225 ms (30 echoes, echo spacing = 9 ms), MTX = 256 x 256, FOV 3.5 x 3.5 cm, 5 slices, 1 mm slice thickness and 1 signal average. Acquisition time was approximately 18 minutes [19].

### Training of segmentation network

**Fig 1:**
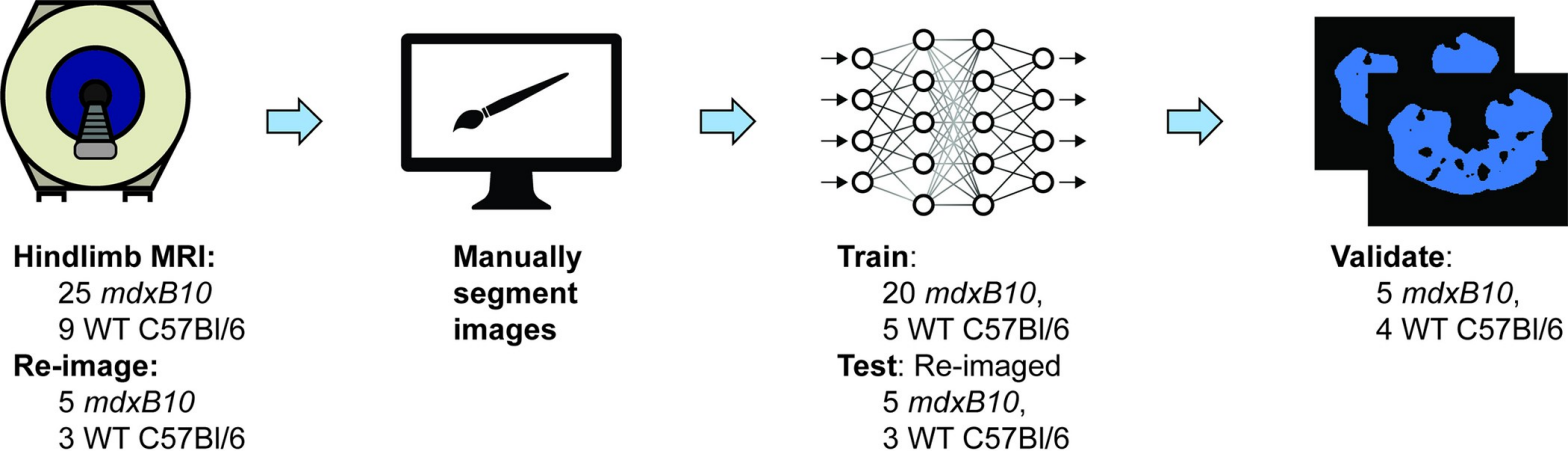
Training of neural network for hindlimb segmentation. Training data was generated from 20 *mdxB10* and 5 WT C57Bl/6 animals. Test data was generated by re-imaging 5 *mdxB10* and 3 WT C57Bl/6 animals. After iterative model refinement, the model was validated with imaging data from an additional 5 *mdxB10* and 4 WT C57Bl/6 animals.

Images were imported into Amira 2020.2 software (Thermo Fisher Scientific, Waltham, MA, USA) and the first echo of the T_2_ map acquisition (TE = 9 ms), a relatively proton-density weighted image, was used to segment a region of interest (ROI) containing hindlimb and paraspinal muscles, and remove other tissues such as bladder, skin, bone, and intermuscular fat. As this was a time-consuming manual process, a deep learning prediction model was trained in Amira using the built-in tools (Fig 1). A training dataset was assembled using segmentation and image data from 20 *mdxB10* scans and 5 WTC57Bl/6 scans, and concatenated with a test dataset assembled from segmentation and image data obtained by rescanning 5 *mdxB10* mice and 3 WTC57Bl/6mice 2–3 days after their initial imaging session. A validation dataset was assembled using data from an additional 5 *mdxB10* mice and 4 WTC57Bl/6 mice. A preliminary model was trained using the “Deep Learning Training” module in Amira with type BackbonedUNet, number of classes = 2, resnet18 backbone, patch size 128, batch size 8, max patch overlap 0.3, maximum number of epochs 100, Adam optimizer, learning rate 0.0001, and no data augmentation. A refined model was trained using the same module, initialized with the weights of the preliminary model, with the following modifications: patch size 256, maximum number of epochs 500, learning rate 0.0005, and data augmentation with geometry transforms including horizontal and vertical flip, 30-degree rotation, 10% zoom, and 10 degree shear, and an early stopping criterion to stop if no model improvement for 25 iterations. The “Segmentation Metrics” Amira Xtra module was used to assess the performance of the model on the validation dataset [20].

### Semiautomated image segmentation

After the deep learning model was generated, the first echo of the T_2_ map acquisition was imported into Amira and segmented using the “Deep Learning Prediction” module using the established model weights. The segmentation was inspected by a trained observer and manually refined as necessary to correct minor errors (e.g., incomplete removal of the bladder). Muscle ROIs were exported from Amira as a mask image to be used in further processing steps. For consistency, all manually segmented datasets used in the study were re-segmented using the semiautomatic segmentation process.

### MR image processing

Using a custom script via the Amira Python console, all echoes of the T_2_ map acquisition were masked to include only muscle voxels using the mask image generated in the segmentation step. The masked data were then imported into JIM 7 (Xinapse Systems Ltd, West Bergholt, Essex, UK) and the apparent T_2_ was fit using the built in nonlinear curve fitting module. The model was monoexponential, of the form:

$$S=S_{0}e^{-\frac{TE}{T_{2}}}$$ Eq 1

where S is the measured signal, S_0_ is the fully recovered signal, TE is the specified echo time, and T_2_ (the quantity being fit) is the transverse relaxation time. A custom Python script was used to extract a list of muscle T_2_ values from the resultant maps. Image slices with significant respiratory artifact were excluded. The top and bottom 1% of T_2_ values were excluded to reduce the effect of outliers on summary statistics. For each set of T_2_ values, the interdecile range (IDR, the difference between the 90^th^ percentile value and the 10^th^ percentile value) was calculated as a measure of distribution spread. The mode (i.e. location of the peak) was computed by fitting a kernel density function to the measured T_2_ values and finding the T_2_ value corresponding to the peak of the distribution. The Pearson Mode Skewness was calculated as a measure of distribution symmetry [21].


$$\frac{mean-mode}{standard\;deviation}$$ Eq 2

### Repeatability of image metrics

To assess the repeatability of measurements of Pearson skew and IDR of the T_2_ distribution, 5 *mdxB10* mice and 3 WT mice were reimaged two days after their initial imaging session. Both sets of data were analyzed according to the protocol described above.

### Evans blue dye

To determine if the MR image-based measures, Pearson skew and IDR, correlate with Evans blue dye uptake measures, *mdxB10*, *mdxD2*, and WT mice were subjected to MR imaging and subsequent evaluation for Evans blue dye uptake in hindlimb muscles (quadriceps, glut/ham, gas/sol). WT mice (n = 5), *mdxB10* mice (n = 4) and *mdxD2* mice (n = 3) underwent the MRI protocol described above followed by measurements of Evans Blue dye uptake that were quantified as described previously [22–24]. Briefly, mice were injected with 5 μl per gram of body weight of 10 μM Evans Blue dye (E2129, Sigma-Aldrich). Mice were euthanized, and tissue was excised, minced, and placed in 1ml of formamide in a 24-well plate placed at 55°C for 2 hrs. Absorbance was measured at 620 nm on a Synergy HTX multi-mode plate reader (BioTek®) from 200 μl of sample solution in a 96-well plate. Each sample was assessed in duplicate. Results are reported as the average arbitrary optical density units per mg of tissue.

### Statistical analysis

A key purpose of this study was to identify and validate relevant summary statistics for MRI image distributions based on the hypothesis that the distribution of T_2_ values would be sufficiently non-normal as to render mean and standard deviation inappropriate summary statistics. A custom Python script was used to test the normality of the distribution of T_2_ values for each animal using D’Agostino’s K2 test, and compute summary statistics [25–29]. All distributions observed were markedly non-normal (p < 0.001). Summary statistics of Pearson mode skew and IDR were investigated to describe the width and asymmetry of distributions and enable quantitative comparisons. Test-retest variability was assessed using Bland-Altman analysis and the comparisons of Pearson skew and IDR with Evans Blue dye uptake were calculated using a simple linear regression model [30].

## Results

### Development of a semi-automated muscle segmentation tool

**Fig 2:**
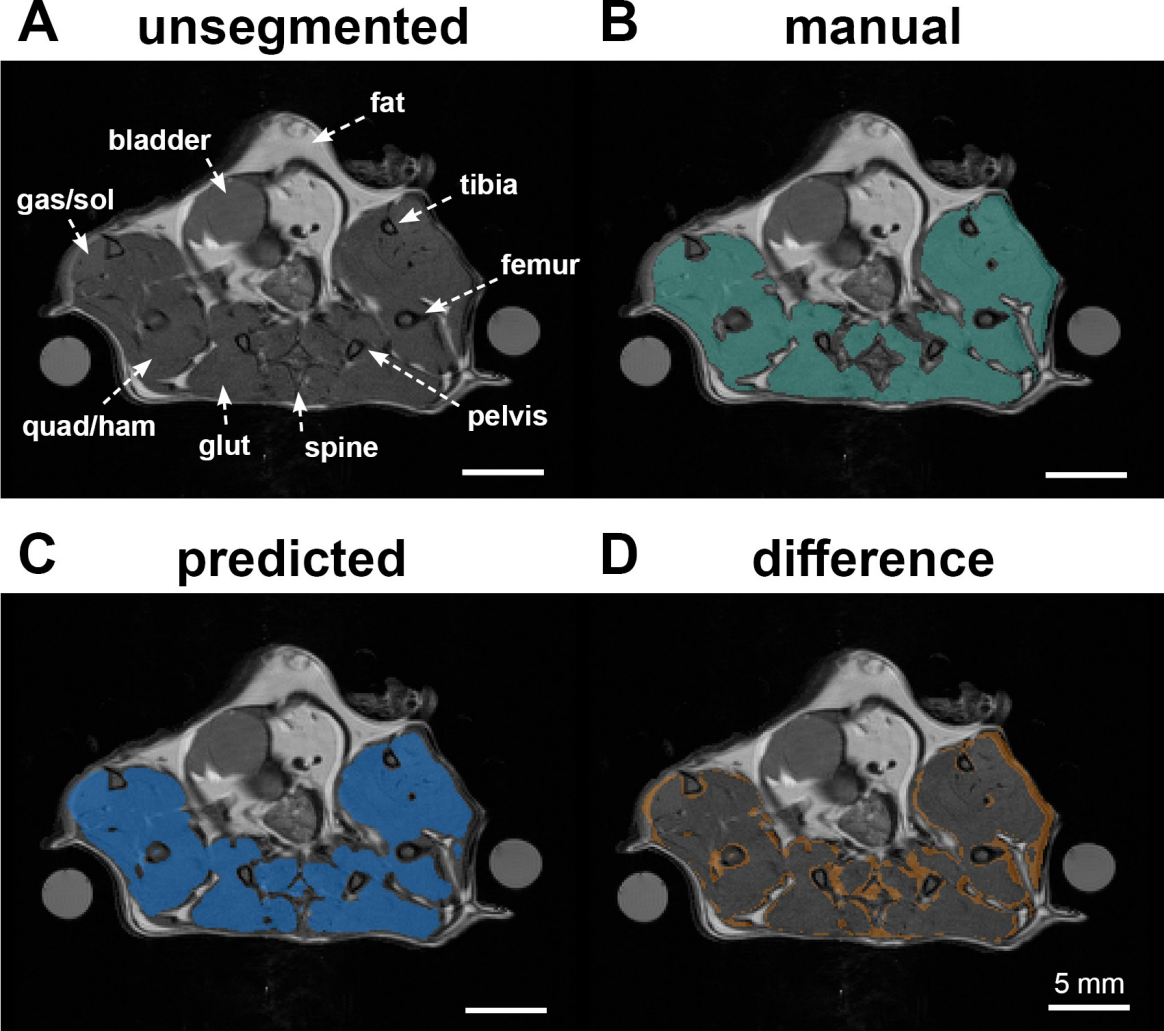
Reliable semi-automatic muscle segmentation. (**A**) Proton density weighted axial image showing hindlimb and paraspinal musculature. Lower hindlimb muscle groups visualized include gastrocnemius and soleus (gas/sol); upper hindlimb muscle groups include quadriceps and hamstrings (quad/ham) and gluteal muscles (glut, near pelvis). (**B**) Manual segmentation of muscle ROI. (**C**) Result of segmentation using the trained deep learning model. (D) Difference between manual and automated segmentation showing overall excellent agreement with minor variation around the muscle-skin and muscle-bone interfaces.

Manual segmentation of the muscle ROI including the hindlimb and paraspinal muscles was a lengthy manual process due to the need to manually separate muscle from other tissues including the peritoneal cavity, testes, bone, and regions of normal fluid and fat found subcutaneously and at intermuscular interfaces. Because of the manual and subjective nature of this process, it was both time-consuming (>1hr per animal) and subject to variability. Use of the deep learning model reduced the time for generating muscle ROIs to a few seconds and reduced total hands-on analyst time to approximately 10 minutes. In the validation dataset, the model-predicted label field matched the ground truth label field with a Jaccard Index of 0.97, indicating excellent agreement. An example segmentation of a dataset not included in the training data is shown in Fig 2.

### Image processing

A total of 39 mice were imaged in 47 imaging sessions. Of the 47 T_2_ maps, one slice was excluded from each of 10 maps due to respiratory artifact, and two slices were excluded from one map. After bounding the masked and T_2_-fitted data to eliminate the bottom 1% and top 1% of T_2_ values for each imaging session, T_2_ values ranged from 19.3–81.4 seconds.

### Stratification of muscle disease through semi-automated MR imaging analysis

**Fig 3:**
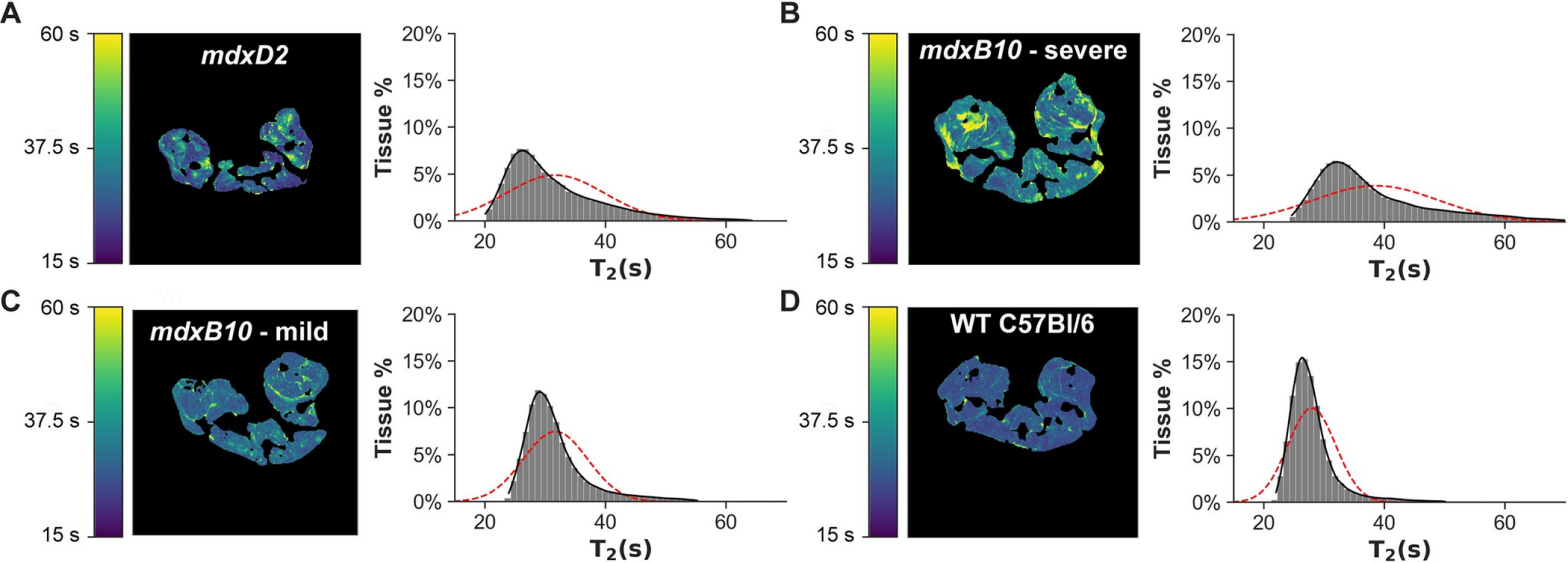
MR image-based measures stratify muscle disease severity. (**A**) Representative image of a *mdxD2* animal with a severe muscle disease phenotype as evidenced by localized areas of high T_2_ corresponding to muscle damage (yellow). (**B**) Representative image of a *mdxB10* animal with a severe disease phenotype seen by an overall increase in T_2_ values combined with distinct regions of elevated T_2_ corresponding to edema. (**C**) Representative image of a *mdxB10* animal with a mild disease phenotype evidenced by a few small focal regions of mildly elevated T_2_ and minimal highly elevated T_2_ voxels. (**D**) Representative image of a healthy WT C57Bl/6 control animal showing minimal regions of high or mildly elevated T_2_ aside from normal intramuscular interfaces. Plots to the right of each image show the histogram of T_2_ values for that animal. The overplotted red dashed lines correspond to the gaussian distribution defined by the mean and standard deviation of the T_2_ distribution, illustrating its inadequacy to describe the actual data.

**Fig 4:**
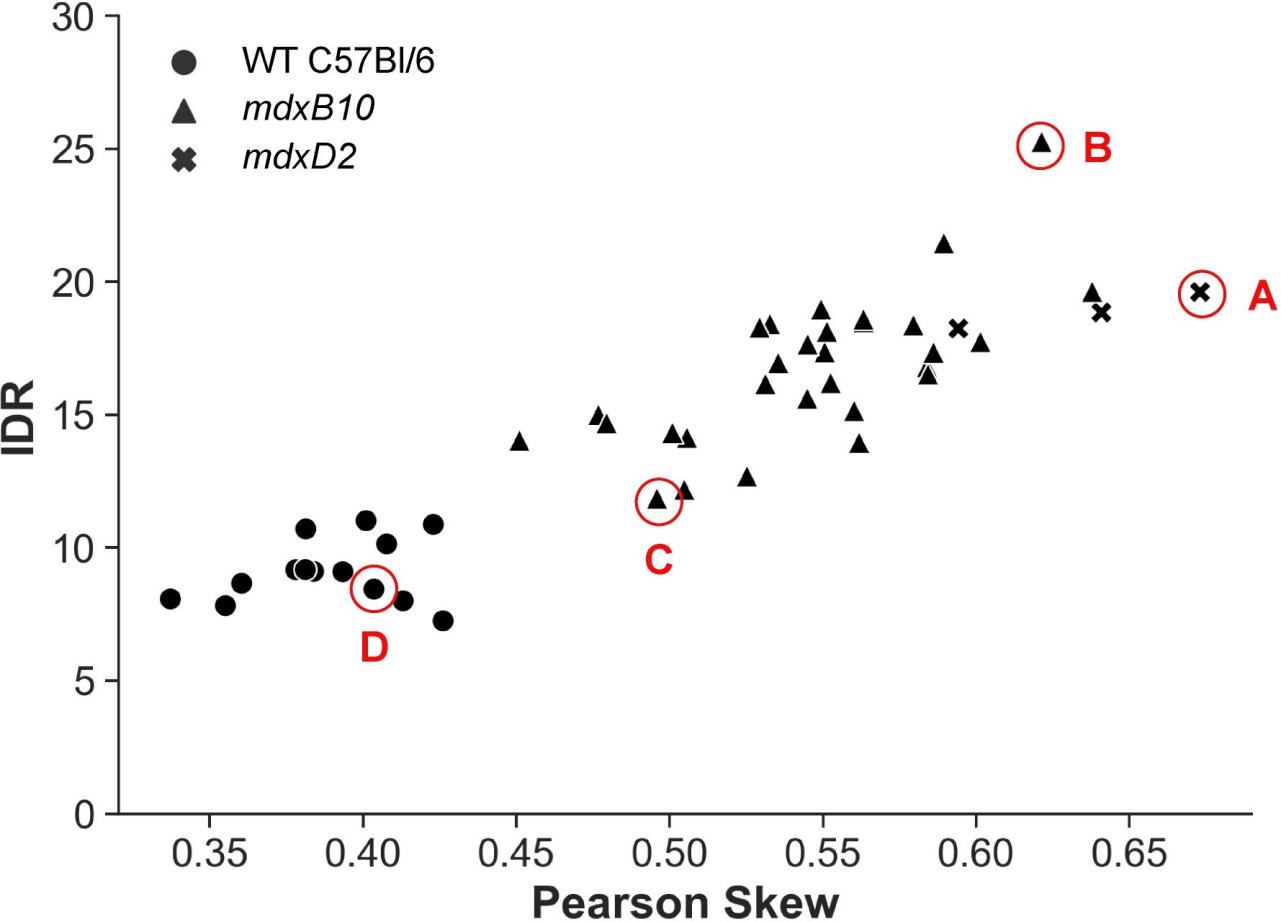
Pearson Skew vs interdecile range (IDR) measures stratify muscle disease severity across mouse models. A plot of Pearson Skew vs IDR for 20 *mdxB10* animals and 13 healthy WT C57Bl/6 control animals, demonstrating that Pearson skew and IDR clearly separate the *mdx* and wildtype animals, identify a spectrum of disease in *mdxB10* animals, and locate *mdxD2* animals at the severe end of the disease spectrum. Data points marked A-D correspond to the images and distributions shown in Fig 3.

The range of disease severity present in this cohort of same-aged and same-sex *mdxB10* mice is evident upon examination of the T_2_ maps shown in Fig 3. Fig 3A illustrates an *mdxD2* mouse, which is known to have a relatively severe muscle disease phenotype. Fig 3B illustrates an *mdxB10* mouse with severe muscle disease as indicated by extensive regions of mildly elevated T_2_, in addition to several sizable regions of highly elevated T_2_. In contrast, Fig 3C illustrates a *mdxB10* mouse with comparatively mild disease, in which there are a few small focal regions with mildly elevated T_2_, but few voxels with highly elevated T_2_. The healthy wildtype mouse shown in Fig 3D has few voxels with high T_2_ and these are primarily located at the intermuscular interfaces. For each T_2_ map, the distribution of T_2_ values is plotted immediately to the right, with the Gaussian distribution corresponding to the data mean and standard deviation overplotted as a dashed line (Fig 3A–3D). In all cases, the distribution of T_2_ values is observed to be non-normal (as confirmed by D’Agostino’s K^2^ test of normality), asymmetric, and poorly characterized by the Gaussian distribution. The shape of the distribution markedly differs between the different animals, with wider, more asymmetric distributions corresponding to more severe disease, and narrower distributions corresponding to milder disease. A plot of Pearson skew vs. IDR of the distribution of T_2_ values was effective in stratifying disease severity and correlated well with visual assessment of the selected T_2_ maps (Fig 4). Together these data show that MR image-based measures extracted using the newly developed, semi-automated segmentation and analysis pipeline stratify muscle disease severity across healthy to severe pathology.

### Semi-automated MR image-based measures are reproducible across disease severity

**Fig 5:**
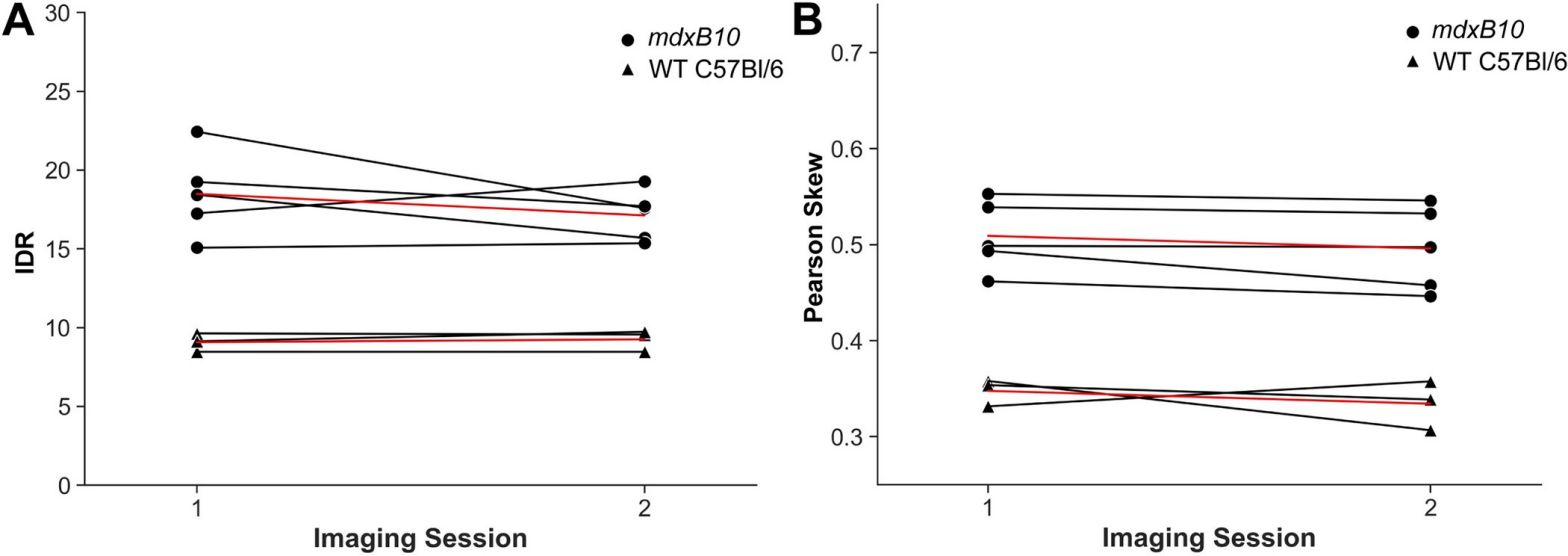
Reproducible MR image-based measures in healthy and diseased muscle. Test-retest reliability comparison of measured values of IDR (**A**) and Pearsons Skew (**B**) in *mdxB10* and WT C57Bl/6 mice imaged twice at an interval of 2 days. Heavy dashed lines represent average values for the two experimental groups.

Both Pearson skew and IDR were found to be highly repeatable across imaging sessions (Fig 5). Based on repeated measures across 5 *mdxB10* and 3 WT C57Bl/6 animals, the minimum detectable change for IDR was 1.75 seconds (12.6% of the average value). The minimum detectable change for Pearson skew was 0.03 (6% of the average value).

### High correlation between measures of muscle membrane leak and semi-automated MR imaging-based measures

**Fig 6:**
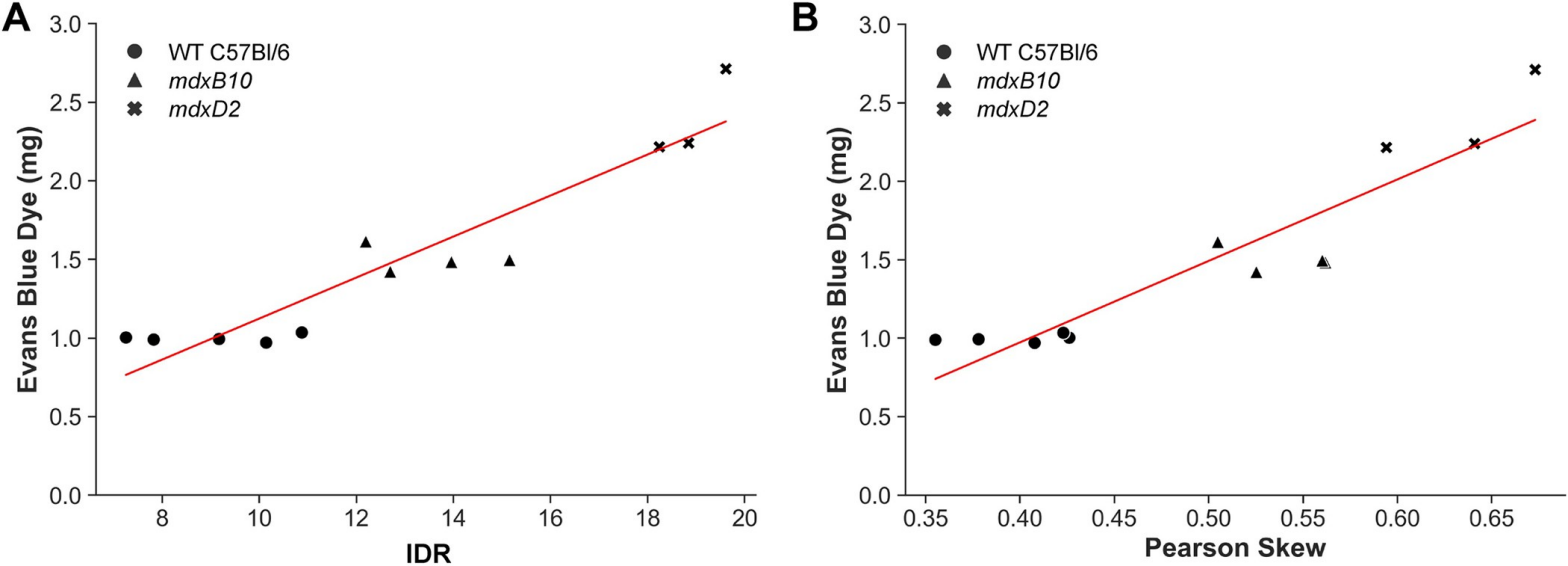
Strong correlation between Evans blue dye uptake and MRI measures across muscle disease severity. Comparison of noninvasive *in vivo* image-based measurements of disease severity IDR (**A**) and Pearsons skew (**B**) with *ex vivo* measurement of Evans blue dye. No muscle disease occurred in healthy WT C57Bl/6 controls (n = 5), mild disease was present in *mdxB10* mice (n = 4), and severe disease was evident in *mdxD2* mice (n = 3). Both measures exhibited a strong linear correlation with the Evans blue dye (R^2^ vs IDR: 0.89; R^2^ vs Pearson skew: 0.86;).

Good agreement was observed between the *in vivo* imaging based metrics (Pearson skew and IDR) measured through the semi-automated MR image segmentation, and the *ex vivo* Evans Blue dye uptake measurement (Fig 6). Based on the Pearson skew and IDR values shown in Fig 4, the *mdxB10* mice used in this experiment had moderate levels of disease. Healthy WT muscle had the lowest level of Evans blue dye uptake and IDR / Pearson skew values followed by *mdxB10*, with *mdxD2* muscle taking up the highest levels of Evans blue dye and having the highest IDR / Pearson skew values. IDR exhibited a strong linear correlation with the Evans blue dye with R^2^ = 0.9 (p < 0.001). Pearson skew was also linearly correlated with R^2^ = 0.71 (p < 0.001). This suggests that the *in vivo* imaging based metrics can serve as a surrogate measure of muscle membrane health and integrity as compared to the terminal Evans blue dye uptake assessment.

## Discussion

We have outlined a non-invasive, quick, and reproducible imaging and analysis protocol based on the distribution of hindlimb T_2_ values that can accurately stratify animals based on disease severity to aid in preclinical therapeutic study design. The associated code for processing and analysis are available online at https://doi.org/10.18131/x17wx-02s47 [31]. Screening animals prior to assigning treatment groups can help mitigate potential confounding effects of the wide range of disease severities in the *mdx* model, as the true treatment effects can be obscured if there are differences in the underlying severity of disease between the groups. Many studies rely on comparing histopathological findings or Evans blue dye uptake in separate groups of untreated and treated animals. Because these measures rely on terminal procedures that cannot be undertaken at the outset of a study, they are especially susceptible to baseline variation between experimental groups.

Elevated T_2_ values reflect regions of muscle disturbance that have previously been correlated with muscle fiber damage in *mdx* mice [17,32,33]. Several previous efforts to use MRI in the assessment of disease severity in *mdx* mice have focused qualitatively on T_2_ weighted images; researchers attempting to use T_2_ maps quantitatively have consistently noted very small differences in the mean value of muscle T_2_ (on the order of a few milliseconds) between *mdx* and wildtype animals with very large standard deviations that confound statistical differentiation between groups [18,34]. We compared the gaussian distribution described by the mean and standard deviation of measured muscle T_2_ values to the actual measured distribution and found that not only was the gaussian distribution a poor descriptor, it masked features useful for stratifying the severity of disease in *mdx* animals. Attempts to differentiate the groups by counting voxels above a specified T_2_ threshold were highly dependent on threshold selection and ineffective at stratifying disease.

Inspection of the distribution of muscle T_2_ values in *mdx* and WT mice identified stark differences in the peak shape and distribution width. All distributions were markedly non-normal, confirming that mean and standard deviation were inappropriate summary statistics. In WT mice, the peak was sharp and narrow, with the mode (location of the peak) occurring at a low T_2_ value corresponding to healthy muscle. The distribution had a shallow tail of higher values corresponding to blood vessels, inter-muscular interfaces, and noise. In contrast, for the *mdxB10* mice, the modes occurred at higher T_2_ values, and the distributions were more asymmetric with heavy tails of high T_2_ values corresponding to regions of edema and necrosis. In *mdxD2* mice, the modes more closely resembled those of WT mice, but the distributions were asymmetric with heavy tails of high T_2_ values corresponding to damaged tissue. Although beyond the scope of this study, further investigation is warranted to elucidate the biological underpinning of the differences between T_2_ distributions observed in *mdxD2* and *mdxB10* mice.

We hypothesized that IDR and Pearson skew would be suitable metrics to assess the width and tail heaviness of T_2_ distributions and therefore the burden of muscle damage. IDR measures distribution spread and captures the magnitude of the highest T_2_ values, and Pearson skew detects a disproportionately high percentage of high-T_2_ values. Since each metric describes a slightly different aspect of the distribution, we plotted them in combination to create a spectrum of disease severity. The metrics clearly and repeatably differentiated *mdx* animals from wildtype animals, as well as differentiating *mdx* animals with severe disease from those with mild disease (Fig 4). Importantly, Pearson skew and IDR correlated well with both qualitative observation of T_2_ weighted MR images and the terminal measurements made using Evans blue dye (Fig 6). Quantification of Evans blue dye uptake has been commonly used to evaluate muscle membrane permeability and stability as a measure of muscle health, as the dye is excluded from healthy intact muscle.

In future studies, this distribution analysis will be extended to a more comprehensive radiomics approach to identify texture-based image features that reflect localized areas of muscle damage [35]. It may be extended to a more in-depth per-muscle analysis, which was explicitly not undertaken in this study as the focus was on developing methods for rapid screening of global animal phenotype severity. A major strength of this rapid semiautomated image segmentation and analysis pipeline is that images of several major hindlimb muscle groups can be acquired in approximately 30 minutes, with only 10–15 minutes per animal needed for semiautomated image segmentation and processing. This significantly increases the throughput relative to manual segmentation and processing, which required 1.5–2 hours per animal, and renders it suitable for screening of cohorts of animals prior to assigning treatment groups. The proposed pipeline could substantially improve the quality of studies of therapeutic efficacy in mouse models of muscular dystrophy by ensuring that study cohorts are appropriately balanced across the spectrum of disease and a priori excluding animals with either extremely severe or extremely mild disease.

## Abbreviations

DMD: Duchenne Muscular Dystrophy
MRI: magnetic resonance imaging

## References

[1] Duan D, Goemans N, Takeda S, Mercuri E, Aartsma-Rus A. Duchenne muscular dystrophy. Nat Rev Dis Primers. 2021;7(1):13. Epub 20210218. doi: 10.1038/s41572-021-00248-3 ; PubMed Central PMCID: PMC10557455. 33602943 PMC10557455

[2] Hoffman EP, Brown RH Jr., Kunkel LM. Dystrophin: the protein product of the Duchenne muscular dystrophy locus. Cell. 1987;51(6):919–28. doi: 10.1016/0092-8674(87)90579-4 . 3319190

[3] Bulfield G, Siller WG, Wight PA, Moore KJ. X chromosome-linked muscular dystrophy (mdx) in the mouse. Proc Natl Acad Sci U S A. 1984;81(4):1189–92. doi: 10.1073/pnas.81.4.1189 ; PubMed Central PMCID: PMC344791. 6583703 PMC344791

[4] Sicinski P, Geng Y, Ryder-Cook AS, Barnard EA, Darlison MG, Barnard PJ. The molecular basis of muscular dystrophy in the mdx mouse: a point mutation. Science. 1989;244(4912):1578–80. doi: 10.1126/science.2662404 . 2662404

[5] McGreevy JW, Hakim CH, McIntosh MA, Duan D. Animal models of Duchenne muscular dystrophy: from basic mechanisms to gene therapy. Disease Models & Mechanisms. 2015;8(3):195–213. doi: 10.1242/dmm.018424 25740330 PMC4348559

[6] Fukada S, Morikawa D, Yamamoto Y, Yoshida T, Sumie N, Yamaguchi M, et al. Genetic background affects properties of satellite cells and mdx phenotypes. Am J Pathol. 2010;176(5):2414–24. Epub 20100319. doi: 10.2353/ajpath.2010.090887 ; PubMed Central PMCID: PMC2861106. 20304955 PMC2861106

[7] Capote J, Kramerova I, Martinez L, Vetrone S, Barton ER, Sweeney HL, et al. Osteopontin ablation ameliorates muscular dystrophy by shifting macrophages to a pro-regenerative phenotype. J Cell Biol. 2016;213(2):275–88. Epub 20160418. doi: 10.1083/jcb.201510086 ; PubMed Central PMCID: PMC5084275. 27091452 PMC5084275

[8] Hammers DW, Hart CC, Matheny MK, Wright LA, Armellini M, Barton ER, et al. The D2.mdx mouse as a preclinical model of the skeletal muscle pathology associated with Duchenne muscular dystrophy. Sci Rep. 2020;10(1):14070. Epub 20200821. doi: 10.1038/s41598-020-70987-y ; PubMed Central PMCID: PMC7442653. 32826942 PMC7442653

[9] Mazala DA, Novak JS, Hogarth MW, Nearing M, Adusumalli P, Tully CB, et al. TGF-beta-driven muscle degeneration and failed regeneration underlie disease onset in a DMD mouse model. JCI Insight. 2020;5(6). Epub 20200326. doi: 10.1172/jci.insight.135703 ; PubMed Central PMCID: PMC7213798. 32213706 PMC7213798

[10] van Putten M, Putker K, Overzier M, Adamzek WA, Pasteuning-Vuhman S, Plomp JJ, et al. Natural disease history of the D2-mdx mouse model for Duchenne muscular dystrophy. FASEB J. 2019;33(7):8110–24. Epub 20190401. doi: 10.1096/fj.201802488R ; PubMed Central PMCID: PMC6593893. 30933664 PMC6593893

[11] Coley WD, Bogdanik L, Vila MC, Yu Q, Van Der Meulen JH, Rayavarapu S, et al. Effect of genetic background on the dystrophic phenotype in mdx mice. Hum Mol Genet. 2016;25(1):130–45. Epub 20151112. doi: 10.1093/hmg/ddv460 ; PubMed Central PMCID: PMC4690497. 26566673 PMC4690497

[12] Beastrom N, Lu H, Macke A, Canan BD, Johnson EK, Penton CM, et al. mdx((5)cv) mice manifest more severe muscle dysfunction and diaphragm force deficits than do mdx Mice. Am J Pathol. 2011;179(5):2464–74. Epub 20110903. doi: 10.1016/j.ajpath.2011.07.009 ; PubMed Central PMCID: PMC3204025. 21893021 PMC3204025

[13] Bencze M, Periou B, Punzon I, Barthelemy I, Taglietti V, Hou C, et al. Receptor interacting protein kinase-3 mediates both myopathy and cardiomyopathy in preclinical animal models of Duchenne muscular dystrophy. J Cachexia Sarcopenia Muscle. 2023;14(6):2520–31. Epub 20231101. doi: 10.1002/jcsm.13265 ; PubMed Central PMCID: PMC10751447. 37909859 PMC10751447

[14] Bronisz-Budzynska I, Chwalenia K, Mucha O, Podkalicka P, Karolina Bukowska S, Jozkowicz A, et al. miR-146a deficiency does not aggravate muscular dystrophy in mdx mice. Skelet Muscle. 2019;9(1):22. Epub 20190814. doi: 10.1186/s13395-019-0207-0 ; PubMed Central PMCID: PMC6693262. 31412923 PMC6693262

[15] Grounds MD, Radley HG, Lynch GS, Nagaraju K, De Luca A. Towards developing standard operating procedures for pre-clinical testing in the mdx mouse model of Duchenne muscular dystrophy. Neurobiol Dis. 2008;31(1):1–19. Epub 20080409. doi: 10.1016/j.nbd.2008.03.008 ; PubMed Central PMCID: PMC2518169. 18499465 PMC2518169

[16] Spurney CF, Gordish-Dressman H, Guerron AD, Sali A, Pandey GS, Rawat R, et al. Preclinical drug trials in the mdx mouse: assessment of reliable and sensitive outcome measures. Muscle Nerve. 2009;39(5):591–602. doi: 10.1002/mus.21211 ; PubMed Central PMCID: PMC4116326. 19260102 PMC4116326

[17] Kobayashi YM, Rader EP, Crawford RW, Campbell KP. Endpoint measures in the mdx mouse relevant for muscular dystrophy pre-clinical studies. Neuromuscul Disord. 2012;22(1):34–42. Epub 20111210. doi: 10.1016/j.nmd.2011.08.001 ; PubMed Central PMCID: PMC3264796. 22154712 PMC3264796

[18] Vohra R, Batra A, Forbes SC, Vandenborne K, Walter GA. Magnetic Resonance Monitoring of Disease Progression in mdx Mice on Different Genetic Backgrounds. Am J Pathol. 2017;187(9):2060–70. doi: 10.1016/j.ajpath.2017.05.010 ; PubMed Central PMCID: PMC5809503. 28826559 PMC5809503

[19] Waters EA. MR imaging of the mouse hindlimb musculature (T2 weighted and T2 map): protocols.io; 2023. Available from: https://protocols.io/view/mr-imaging-of-the-mouse-hindlimb-musculature-t2-we-cucjwsun.

[20] Image Segmentation Evaluation Using Standard Metrics (Python): Thermo Fisher Scientific; 2021 [updated March 8, 2021; cited 2023 May 1, 2023]. Available from: https://xtras.amira-avizo.com/xtras/image-segmentation-evaluation-using-standard-metrics-python.

[21] Weisstein EW. Pearson Mode Skewness MathWorld—A Wolfram Web Resource: Mathworld; [10/9/2023]. Available from: https://mathworld.wolfram.com/PearsonModeSkewness.html.

[22] Heydemann A, Ceco E, Lim JE, Hadhazy M, Ryder P, Moran JL, et al. Latent TGF-beta-binding protein 4 modifies muscular dystrophy in mice. J Clin Invest. 2009;119(12):3703–12. Epub 20091102. doi: 10.1172/JCI39845 ; PubMed Central PMCID: PMC2786802. 19884661 PMC2786802

[23] Demonbreun AR, Wyatt EJ, Fallon KS, Oosterbaan CC, Page PG, Hadhazy M, et al. A gene-edited mouse model of limb-girdle muscular dystrophy 2C for testing exon skipping. Dis Model Mech. 2019;13(2). Epub 20191104. doi: 10.1242/dmm.040832 ; PubMed Central PMCID: PMC6906631. 31582396 PMC6906631

[24] Demonbreun AR, Fallon KS, Oosterbaan CC, Bogdanovic E, Warner JL, Sell JJ, et al. Recombinant annexin A6 promotes membrane repair and protects against muscle injury. J Clin Invest. 2019;129(11):4657–70. doi: 10.1172/JCI128840 ; PubMed Central PMCID: PMC6819108. 31545299 PMC6819108

[25] Virtanen P, Gommers R, Oliphant TE, Haberland M, Reddy T, Cournapeau D, et al. SciPy 1.0: fundamental algorithms for scientific computing in Python. Nature Methods. 2020;17(3):261–72. doi: 10.1038/s41592-019-0686-2 32015543 PMC7056644

[26] Pedregosa F, Varoquaux G, Gramfort A, Michel V, Thirion B, Grisel O, et al. Scikit-learn: Machine Learning in Python. Journal of Machine Learning Research. 2011;12(85):2825–30.

[27] McKinney W. Data Structures for Statistical Computing in Python. In: van der Walt S, Millman J, editors. Proceedings of the 9th Python in Science Conference; 20102010. p. 56–61.

[28] Hunter JD. Matplotlib: A 2D Graphics Environment. Computing in Science & Engineering. 2007;9(3):90–5. doi: 10.1109/MCSE.2007.55

[29] Harris CR, Millman KJ, van der Walt SJ, Gommers R, Virtanen P, Cournapeau D, et al. Array programming with NumPy. Nature. 2020;585(7825):357–62. doi: 10.1038/s41586-020-2649-2 32939066 PMC7759461

[30] Bland JM, Altman DG. Measuring agreement in method comparison studies. Statistical Methods in Medical Research. 1999;8(2):135–60. doi: 10.1177/096228029900800204 10501650

[31] Waters EA. Waters Demonbreun MDX MRI Manuscript Data Package. 1.0.0 ed. Prism: Galter Health Sciences Library, Northwestern University; 2023. 10.18131/x17wx-02s47.

[32] Walter G, Cordier L, Bloy D, Lee Sweeney H. Noninvasive monitoring of gene correction in dystrophic muscle. Magnetic Resonance in Medicine. 2005;54(6):1369–76. doi: 10.1002/mrm.20721 16261578

[33] Mathur S, Vohra RS, Germain SA, Forbes S, Bryant ND, Vandenborne K, et al. Changes in muscle T2 and tissue damage after downhill running in mdx Mice. Muscle & Nerve. 2011;43(6):878–86. doi: 10.1002/mus.21986 21488051 PMC3101319

[34] Dunn JF, Zaim-Wadghiri Y. Quantitative magnetic resonance imaging of the mdx mouse model of Duchenne muscular dystrophy. Muscle Nerve. 1999;22(10):1367–71. doi: 10.1002/(sici)1097-4598(199910)22:10&lt;1367::aid-mus5&gt;3.0.co;2-h . 10487902

[35] Apte AP, Iyer A, Crispin-Ortuzar M, Pandya R, van Dijk LV, Spezi E, et al. Technical Note: Extension of CERR for computational radiomics: A comprehensive MATLAB platform for reproducible radiomics research. Med Phys. 2018. Epub 20180613. doi: 10.1002/mp.13046 ; PubMed Central PMCID: PMC6597320. 29896896 PMC6597320

